# Rationale and design of the Henan ST elevation myocardial infarction (STEMI) registry: a regional STEMI project in predominantly rural central China

**DOI:** 10.1186/s12872-019-1250-9

**Published:** 2019-11-28

**Authors:** You Zhang, Shan Wang, Shuyan Yang, Shanshan Yin, Qianqian Cheng, Muwei Li, Datun Qi, Xianpei Wang, Zhongyu Zhu, Luosha Zhao, Dayi Hu, Chuanyu Gao

**Affiliations:** 1 Department of Cardiology, Zhengzhou University People’s Hospital, No. 1 Fuwai Road, Zhengzhou, 450018 Henan China; 2 Department of Cardiology, Henan Provincial People’s Hospital, Zhengzhou, Henan China; 3 Department of Cardiology, Central China Fuwai Hospital, Zhengzhou, Henan China; 4 Henan Institute of Cardiovascular Epidemiology, Zhengzhou, Henan China; 5 Department of Electrocardiography, Henan Chest Hospital, Zhengzhou, Henan China; 6 Henan Academy of Medical Sciences, Zhengzhou, Henan China; 7 Department of Cardiology, the First Affiliated Hospital of Zhengzhou University, Zhengzhou, Henan China; 8 Heart centre, Peking University People’s Hospital, Beijing, China

**Keywords:** ST elevation myocardial infarction, Rural health, Mortality, Quality improvement, Registry, China

## Abstract

**Background:**

Cardiovascular disease including ST elevation myocardial infarction (STEMI) is increasing and the leading cause of death in China. There has been limited data available to characterize STEMI management and outcomes in rural areas of China. The Henan STEMI Registry is a regional STEMI project with the objectives to timely obtain real-world knowledge about STEMI patients in secondary and tertiary hospitals and to provide a platform for care quality improvement efforts in predominantly rural central China.

**Methods:**

The Henan STEMI Registry is a multicentre, prospective and observational study for STEMI patients. The registry includes 66 participating hospitals (50 secondary hospitals; 16 tertiary hospitals) that cover 15 prefectures and one city direct-controlled by the province in Henan province. Patients were consecutively enrolled with a primary diagnosis of STEMI within 30 days of symptom onset. Clinical treatments, outcomes and cost are collected by local investigators and captured electronically, with a standardized set of variables and standard definitions, and rigorous data quality control. Post-discharge patient follow-up to 1 year is planned. As of August 2018, the Henan STEMI Registry has enrolled 5479 patients of STEMI.

**Discussion:**

The Henan STEMI Registry represents the largest Chinese regional platform for clinical research and care quality improvement for STEMI. The board inclusion of secondary hospitals in Henan province will allow for the exploration of STEMI in predominantly rural central China.

**Trial registration:**

[NCT02641262] [29 December, 2015].

## Background

With the effect of changing lifestyles and an aging population, prevalence of cardiovascular disease is increasing in China, with estimates of 290 million individuals being affected by the disease [1, 2]. Nowadays cardiovascular disease is the leading cause of death in China [3–5], and cardiovascular mortality in rural China has been higher than that of urban areas since 2009 [1]. ST elevation myocardial infarction (STEMI) is one of the most severe cardiovascular diseases. Though new interventional techniques in percutaneous coronary intervention (PCI) and new guidelines-recommend drugs have been developed in last 20 years, large gap existed between clinical practice and guideline recommendation [6, 7].

The China Patient-centered Evaluative Assessment of Cardiac Events Retrospective Study of Acute Myocardial Infarction (China PEACE-Retrospective AMI Study) which included 13,815 patients with STEMI from 162 hospitals over 3 years (2001, 2006, and 2011), found that the administration of PCI was increasing in China, and estimated national rates of hospitalizations for PCI had increased by 21-fold from 2001 to 2011 [8]. However, there was no change in the proportion of primary PCI (approximately 30%), early reperfusion and in-hospital death in 2001–2011 [7]. China Acute Myocardial Infarction (CAMI) registry showed that symptom-onset-to-balloon time in patients with STEMI undergoing primary PCI was longer than that in registry studies from other countries [9].

Henan is the most populated (94.4 million in 2015) and predominantly rural (55%) province in central China [10], with a high burden of cardiovascular disease [5]. Our survey conducted in 2012 showed that only 35.1% of STEMI patients received early reperfusion, and fibriolysis was the primary reperfusion therapy (29.2%). In-hospital mortality was 5.8%. Moreover, for patients who didn’t receive reperfusion, doctor’s decision (29%), missed 12-h window (26%), patient refusal (20.4%), and age more than 75 years (15.5%) were main reasons [11, 12].

Based on the results of China PEACE-Retrospective AMI Study, China has begun to place more emphasis on STEMI care. In 2013, the chest pain centre (CPC) certification system just began to be established, and three national multicentre clinical trials on STEMI were initiated in 2011–2014: (1) The China STEMI Care Project, aimed to increase the early reperfusion use by establishment of regional STEMI care network, was initiated in 15 provinces including Henan [13]; (2) The Improving Care for Cardiovascular Disease in China – Acute Coronary Syndrome (CCC-ACS) project, a national hospital-based quality improvement program, aimed to increase adherence to ACS guidelines in China and improve patient outcomes, enrolled 150 tertiary centres across China [14]; (3) The CAMI registry, the largest national long-term registry-research-education platform for surveillance, research, prevention and care improvement for AMI in China, including provincial, prefectural and county-level hospitals [15]; All these studies were conducted in Henan province. In the last 5 years, 21 regional STEMI care networks have been initially developed in Henan. And the number of CPCs has been increased from 6 (1 secondary hospital, 5 tertiary hospitals) in 2016 to 43 (13 secondary hospitals, 30 tertiary hospitals) in 2018. The STEMI promotional videos are broadcast on major television stations and public welfare propaganda on STEMI treatment such as Myocardial Infarction Care Day on November 20th, is held to let the public know call 120 on case of chest-pain onset, and open the occluded coronary artery within 120 min of symptom onset.

However, it is unknown whether the reperfusion rate and mortality of STEMI are improving after the above improvements in central China. Until now, there is no representative long-term registry program for the patients with STEMI in predominantly rural areas of China. Therefore, we designed and launched Henan STEMI registry, a regional registry of the hospitalized patients with STEMI as an integrated research and hospital-based quality improvement platform.

The overall objective of Henan STEMI registry is to timely obtain real-world knowledge about STEMI patients and to provide the platform for care quality improvement efforts in central China. The specific aims of Henan STEMI registry are to: (1) assess management practices, time delays, outcomes in consecutive STEMI patients in reperfusion-capable hospitals, i.e., tertiary and secondary, in Henan province; (2) find gaps between clinical practice and guideline; (3) improve the use of evidence-based guidelines through the feedback of the key performance indicators (KPIs).

## Methods and design

### Study design

This is a multicentre, prospective and observational study for STEMI patients, which is aimed to collect all clinical information related to symptom onset, diagnosis, treatment and prognosis. STEMI patients within 30 days of symptom onset in secondary and tertiary hospitals were consecutively enrolled, and followed within 1 month, 6 months and 12 months of symptom onset.

**Fig. 1.**
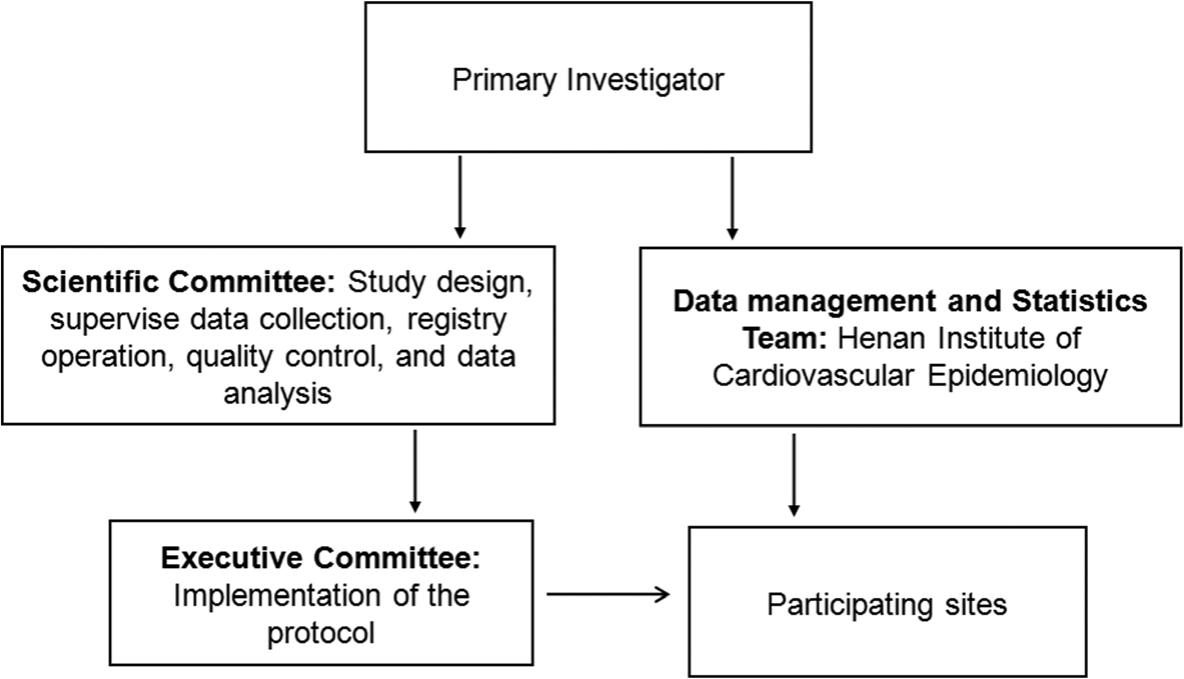
Organizational framework of the Henan STEMI registry

The registry is organized and conducted by Zhengzhou University People’s Hospital. The organizational structure of Henan STEMI registry is displayed in Fig. 1. The principal investigator (PI) is responsible for all aspects of the registry, including the study design, conduct, and report of the registry. The Scientific Committee composed of PI and academic experts are responsible to design the registry, supervise data collection, registry operation, quality control, and data analysis. The members of Executive Committee consist of PIs of each participating sites, and manage the execution of the registry. Henan Institute of Cardiovascular Epidemiology (Henan ICE) is in charge of data quality evaluation, data checking, analysis and management. Each local PI appoints a research coordinator to help investigators enroll the patients, collect and submit data.

### Site selection

According to the measures for the hospital classification management by the Chinese government, hospitals are classified into three grades. Primary hospitals are community hospitals or township health centres with only the most basic facilities and very limited resources to serve inpatients, and do not provide reperfusion therapy. Secondary hospitals have at least 100 inpatient beds and the ability to provide acute medical care and preventive care services to populations of at least 100,000. Tertiary hospitals are major referral centres in provincial capitals and major cities. Therefore, secondary and tertiary hospitals as reperfusion-capable hospitals were included in this STEMI registry.

There are five practical levels of local government: province, prefecture, county, township, and village, according to Chinese administrative divisions. And public hospitals were founded following this structure and provide the majority of health care. Totally, there are 17 prefectures and a city direct-controlled by the province in Henan province, and in each of prefectures there are some counties. The Henan STEMI registry included one provincial-level academic hospital (tertiary hospital); and in every prefecture, this registry included one to three prefectural-level hospitals (tertiary hospitals) and 1 to 10 county-level hospitals (secondary hospitals). If the local PI was willing to participate in the project, the site would be included. These three levels of hospitals reflect typical Chinese governmental and administrative model in China.

**Fig. 2.**
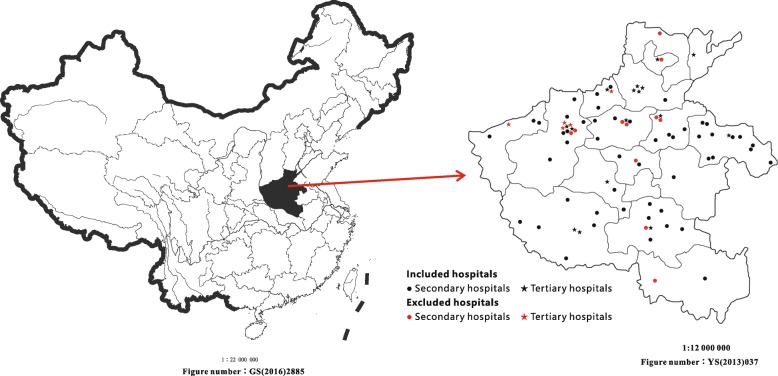
Geographic distribution of hospitals. The China map manufactured by the supervision of National Administration of Surveying, Mapping and Geoinformation. Two maps were both free downloaded from the website of Henan Administration of Surveying, Mapping and Geoinformation (http://www.hnch.gov.cn/plus/view.php?aid=7534).

Totally, with 16 hospitals excluded due to non-compliance with continuous enrollment or no patients enrollment, there are 66 participating hospitals (50 secondary hospitals; 16 tertiary hospitals) that cover 15 prefectures and one city direct-controlled by the province in Henan province, which is to assure a broad representation of hospitals across different levels, with broad coverage of geographic region, rural and urban areas of Henan (Table 1, Fig. 2 and Additional file 1).

**Table 1:** Characteristics of participating hospitals

	Secondary hospital	Tertiary hospital
Number of hospitals	50	16
Number of beds in cardiology	60 (47, 96)	143 (106, 217)
CCU	40 (80%)	16 (100%)
Catheter labs	42 (84%)	16 (100%)

*CCU* Coronary care unit

### Patient population

#### Inclusion and exclusion criteria

Patients whose primary diagnosis was STEMI within 30 days of symptom onset were admitted consecutively according to the number of patients scheduled to be enrolled in the agreement signed with each sub-centre, and enrollment could be stopped if the centre completed the target number of cases.

STEMI was defined in accordance with the universal definition of Myocardial Infarction (2012) [16], specifically as persistent ST-segment elevation (≥ 0.1 mV at J points) in 2 or more contiguous leads or new onset of left bundle branch block. Furthermore, according to the classification of myocardial infarction, types 1, 2, and 3 and types 4b and 4c are included in the present registry. Types 4a and type 5 are excluded from the Henan STEMI registry.

#### Data collection

Clinical data are collected via a secure, password-protected, web-based data collection platform developed by Zhao Rui Corporation (http://117.159.29.146:18999). Each hospital is responsible for its own data collection and reporting, and trained investigators enter the data elements abstracted from medical records. Data elements collected include patient demographics, medical histories, risk factors, pre-hospital information, laboratory and imaging results, in-hospital treatment, reperfusion strategies, medications, clinical events, cost and follow-up information (1-month, 6-month and 1-year) (Table 2). Each patient will be assigned a unique ID identified through patient ID number to ensure no duplicate data input and be used to data query and revision and track post-discharge follow-up. Site investigators are required to collect all the data during the hospitalization and submit the completed electronic case report form upon the patient’s discharge or death. Face-to-face interview or telephone follow-up visits are planned at 1 month, 6 months and 1 year after STEMI onset. The data collection platform automatically reminds investigators of follow-up.

**Table 2:** The Henan STEMI Registry data elements

Category	Example elements
Demographics	Date of birth, sex, ethnicity, height, weight, marital status, occupation, education level, and medical insurance status
Medical history and risk factors	Hypertension, hyperlipidemia, diabetes, smoking, prior stroke, family history of coronary heart disease, prior angina, prior MI, prior heart failure, prior revascularization, peripheral artery disease, chronic kidney disease, COPD, peptic ulcer and bleeding
Pre-hospital information	Patient (symptom onset date/time, symptoms, cardiac arrest) First-contact hospital (hospital approaching method, first medical contact time, first ECG time, consent time, reperfusion strategies, DI-DO time, and transfer process)
In-hospital treatment	Department to arrive, first ECG date/time and findings, cardiac function classification, admission blood pressure, heart rate, infarct location, and Killip class Reperfusion strategies, thrombolysis (time, thrombolytic agent, dose and outcome), primary and rescue PCI (time, coronary angiography results, culprit vessels, diameter and length of stent, TIMI flow before and after PCI, residual stenosis), and reasons why patients did not receive early reperfusion therapy Treatment delay (hospital approaching method, first medical contact time, first ECG time, catheter lab ready time, consent time, FMC2B, FMC2N, and reasons of delay)
Laboratory results	Troponin, CK-MB, CRP, BNP, NT-proBNP, lipids, hepatic and renal function, fasting glucose, HbA1c, blood routine test, ECG and UCG
Medications	Antiplatelets, heparin, statin, β-blocker, ACEI or ARB, other medications used 1 week before MI onset, during hospitalization and at discharge
At discharge	Diagnosis, in-hospital duration, expense, reason for discharge, medication and MACCE (Death, reinfarction, in-stent thrombosis, heart failure, cardiogenic shock, cardiac arrest, mechanical complications, ischemic stroke, revascularization and bleeding)
Follow-up	Presentation status (symptom, cardiac function classification, and smoking), medication, laboratory results and MACCE (death, reinfarction, in-stent thrombosis, congestive heart failure, rehospitalization, ischemic stroke, revascularization and bleeding)

*ACEI* Angiotensin-converting enzyme inhibitor, *ARB* Angiotensin receptor blocker, *BNP* Brain natriuretic peptide, *CK-MB* Creatine kinase MB isoenzyme, *COPD* Chronic obstructive pulmonary disease, *CRP* C-reactive protein, *DI–DO* Door in–door out, *ECG* Electrocardiograph, *FMC2B* First medical contact to balloon, *FMC2N* First medical contact to needle injection, *HbA1c* Glycosylated hemoglobin A1c, *MACCE* Adverse cardiovascular and cerebrovascular events, *MI* Myocardial infarction, *NT-ProBNP* N-terminal pro–brain natriuretic peptide, *PCI* Percutaneous coronary intervention, *UCG* Ultrasound cardiogram

#### Data management and quality control

The kick-off investigator meeting and first training workshop were held on 26 August, 2016. At this meeting, all investigators received detailed training on the protocol, standard definitions of the elements, web-based software system, data collection and data entry procedures. And a We Chat group was established to facilitate communication and solve problems related to the study.

Data accuracy and completeness of Henan STEMI registry are mainly ensured by the following 6 strategies: 1) Diagnosis of STEMI is according to the third universal definition of myocardial infarction; 2) Before registry, a training program on study objectives, data collection, and STEMI management is given to the primary investigator and related staff at each participating centre; 3) Real-time automatic logic and range check on the completeness and validity of the data are integrated in the data entry system to control basic data quality. Henan ICE regularly provides data quality checks and sends queries for illogical, invalid or missing data elements to participating hospitals to review and revise. 4) Participating centres who have the high error rate of data, and no response to the queries in 6 months shall be deemed abandoned automatically; participating centres who have the high quality of data will be issued certificates to reward; 5) Henan ICE checks the consecutiveness of all cases in every participating site, and regularly monitors at least 10% of reported cases for accuracy against medical records randomly. If the reported cases are not completed with 98% accuracy, all reported cases are considered unqualified and this investigator will be retrained; 6) Investigator meeting will be annually held to conclude the progress, solve existing problems and strengthen program training.

#### Study outcomes

The primary outcome in this study is in-hospital all-cause mortality. The secondary outcomes include all-cause mortality in 1 month, 6 month and 12 month, and main adverse cardiovascular and cerebrovascular events (MACCE, death, congestive heart failure, reinfarction and ischemic stroke) during the hospitalization.

#### Quality of care assessment

In Henan STEMI registry, performance measures were developed to measure the quality of care for STEMI patients based on the class I recommendation of guidelines for STEMI [17]. The KPIs presented in the monthly reports consist of primary and secondary performance measures. There are 18 primary performance measures, including 7 for acute treatment (aspirin at arrival, P2Y12 inhibitor at arrival, arrival by emergency ambulance, early reperfusion therapy, time to fibrinolysis, time to primary PCI, and evaluation of left ventricular function), 8 performance measures at discharge (aspirin at discharge, P2Y12 inhibitor at discharge, statin at discharge, β-blocker at discharge, angiotensin-converting enzyme inhibitor or angiotensin receptor blocker at discharge, in-hospital all-cause mortality, in-hospital death or treatment withdrawal, and in-hospital MACCE), and 3 performance measures for follow-up. There are 7 secondary performance measures (Table 3).

**Table 3:** KPIs for the Henan STEMI registry

Primary performance measures	
During hospitalization	
Proportion of patients receiving aspirin at arrival Proportion of patients receiving P2Y12 inhibitor at arrival Proportion of patients arrival by emergency ambulance Proportion of patients receiving early reperfusion therapy Proportion of patients receiving fibrinolytic therapy within 30 min of first medical contact among those receiving this treatment Proportion of patients receiving primary PCI within 90 min of first medical contact among those receiving this treatment Proportion of patients with evaluation of left ventricular function by echocardiography	
At discharge	
Proportion of patients receiving aspirin at discharge Proportion of patients receiving P2Y12 inhibitor at discharge Proportion of patients receiving statin at discharge Proportion of patients with indications receiving a β-blocker at discharge Proportion of patients with indications receiving an ACEI or ARB at discharge In-hospital all-cause mortality In-hospital death or treatment withdrawal In-hospital MACCE (death, congestive heart failure, reinfarction and ischemic stroke)	
Follow-up	
Proportion of patients receiving 1-month on-time follow-up Proportion of patients receiving 6-month on-time follow-up Proportion of patients receiving 1-year on-time follow-up	
Secondary performance measures	
Proportion of patients receiving first ECG within 10 min of first medical contact Proportion of delayed primary PCI (Time from first medical contact to primary PCI > 90 min) Proportion of patients receiving anticoagulants at arrival (within 24 h) Proportion of patients receiving determination of blood LDL level Proportion of patients receiving P2Y12 inhibitor at discharge among those receiving reperfusion Proportion of patients receiving P2Y12 inhibitor at discharge among those without receiving reperfusion Proportion of patients receiving aspirin and P2Y12 inhibitor at discharge	

*ACEI* Angiotensin-converting enzyme inhibitor, *ARB* Angiotensin receptor blocker, *ECG* Electrocardiograph, *LDL* Low density lipoprotein, *MACCE* Main adverse cardiovascular and cerebrovascular events, *PCI* Percutaneous coronary intervention

Hospitals receive monthly quality reports on the KPIs, summarized and compared against a variety of internal and external benchmarks. Internal benchmarks include the trend of performance measures over time. External benchmarks are intended to provide reasonable performance thresholds of all participating sites to help identify areas for potential improvement of care quality. Hospitals can download the reports from the online platform, accessed with a username and password.

#### Sample size

The sample size determination for the study is based on the previous survey data [11]. We determined the sample size for describing the primary outcome, in-hospital mortality, which we had estimated to be ≈6.0% in secondary hospitals and 5.6% in tertiary hospitals. To achieve a power of 80% with a two-sided type I error of 0.05, we estimated a requested sample size of 2500 for each class of hospital.

#### Statistical analysis

Investigators will report summary statistics for patient characteristics, delay time (symptom onset-to-first medical contact (FMC), FMC-to-reperfusion), patient referral (time and means of transportation), treatments received and primary outcomes, and compare these indexes between secondary and tertiary hospitals. The prespecified subgroup analyses mainly include but are not limited to age, gender, Killip class, time to first medical contact, infarct location, history of hypertension, diabetes or smoking and thrombolysis in myocardial infarction risk score. Statistical analyses are performed using SAS 9.4 (SAS Institute, Cary, NC, USA).

#### Trial status

The first participant was enrolled in September 2016. As of August 2018, 5479 patients of STEMI have been enrolled and recruitment has been stopped. Follow-up is planned to continue until September 2019.

## Discussion

Henan STEMI registry is a regional platform for clinical research and care quality improvement on STEMI. This registry is helpful to get the knowledge of real-world treatment and outcome of patients with STEMI in rural central China, to expand implementation of the guidelines though the feedback of KPIs, and moreover, to provide scientific data for policy making.

China, with its vast landmass and large population, has the unbalanced economic development. Henan, located in central China, is a microcosm of China’s economy. However, limited data is available on the treatment status of STEMI in the rural areas besides national studies [6, 18]. Henan STEMI registry provides a chance to fully understand the real-world management and long-term outcome of STEMI patients in rural China. Only tertiary hospitals were included in the China STEMI care program and CCC-ACS projects, and 6 secondary hospitals in 10 hospitals in Henan province participated in CAMI registry. Whereas 50 secondary hospitals are included in Henan STEMI registry, therefore Henan STEMI registry is the largest STEMI registry in rural areas of China.

As a big difference on medical care resources of AMI exists between different-level hospitals [19], comparison between secondary and tertiary hospitals in Henan STEMI registry would summarize the gaps between the current status of hospital treatment at different levels and current guidelines, analyze the influence factors of not receiving early reperfusion therapy and mortality, and develop measures to promote early reperfusion therapy and reduce mortality. We undertook a survey on the reperfusion treatment and in-hospital outcome of STEMI patients in 2011–2012, so comparison between different time periods could better illustrate the improvement and summarize appropriate improvement strategies in rural areas. Meanwhile, we would make comparison of relevant studies at home and abroad. Subgroup analyses in age [18, 20, 21], sex [22, 23], heart function [24], time to first medical contact [25, 26], infarct location, complicated with hypertension, diabetes or smoking [27], and risk score would be made to provide more information for cardiologists to pay attention to special populations and reduce treatment differences. Moreover, risk estimation model would be explored to identify risk of in-hospital mortality in rural areas [28, 29]. This registry could contribute important scientific questions for further clinical research.

Henan STEMI registry is also a platform for care quality improvement on STEMI in predominantly rural central China. In the last 10 years, there has been great progress in the treatment of STEMI, such as PCI and lots of guidelines-recommend drugs. However, secondary hospitals often has limited resources [19], for instance, lack of interventional cardiologists and equipment, and fewer opportunities for guideline training, whereas they are always the hospitals where the first medical contact of rural STEMI patients are made, so care quality improvement in rural areas is an urgent need. We use the data of Henan STEMI registry to give the feedback of KPIs to help cardiologists get the knowledge of the gap between practice and guidelines, and further enhance their consciousness to give the guideline-recommend treatment. Moreover, most tertiary hospitals in this registry, participated in more national care improvement projects, play a significant exemplary role on secondary hospitals.

During the Henan STEMI registry, patients get a chance to get full knowledge of the STEMI, choose the proper healthy lifestyle, and keep better compliance to drug treatments. And the administrative departments of hospitals or health authorities could improve the system quality of care for patients with STEMI through the results of the registry.

The Henan STEMI registry is distinguished that it is a large multicentre, prospective rural STEMI registry, which is also platform for care quality improvement on STEMI. These help to comprehensively get the knowledge of real-world practice care, outcomes, and cost in rural central China, and facilitate the translation of study findings to improvement of quality care for STEMI in rural areas. In this registry, a one-year follow-up is given to dynamically observe the change of medication use, care, and outcomes for patients with STEMI over time. Costs during hospitalization are also collected to analyze cost-effectiveness and burden for STEMI patients.

There are several limitations that should be considered. Two prefectures named Luohe and Anyang did not participate in this registry for various reasons. However, we believe our registry will reflect the routine clinical practice of STEMI care in central China for the representativeness of other 16 prefectures included in our registry. In addition, the data collection burden for investigators may be the greatest barrier to the registry that may lead to some enrollment bias. We have carefully considered each element to limit the burden and have quality control measures in the registry.

## Conclusions

This registry represents the largest Chinese regional platform for clinical research and care quality improvement for STEMI. The board inclusion of secondary hospitals in Henan province will allow for the exploration of STEMI in predominantly rural central China. The results may be useful for cardiologists and police-makers in the world-wide rural areas.

## Supplementary information

**Additional file 1.** Full list of hospitals in the Henan STEMI registry

## Data Availability

The datasets generated and/or analyzed during the current study are available from the corresponding author on reasonable request.

## Abbreviations

CAMI registry: China acute myocardial infarction registry
CCC-ACS project: The improving Care for Cardiovascular Disease in China – acute coronary syndrome project
China PEACE-Retrospective AMI Study: The China patient-centered evaluative assessment of cardiac events retrospective study of acute myocardial infarction study
CPC: Chest pain Centre
FMC: First medical contact
Henan ICE: Henan institute of cardiovascular epidemiology
KPIs: Key performance indicators
MACCE: Main adverse cardiovascular and cerebrovascular events
PCI: Percutaneous coronary intervention
PI: Principal investigator
STEMI: ST elevation myocardial infarction

## References

[1] Weiwei C, Runlin G, Lisheng L, Manlu Z, Wen W, Yongjun W, Zhaosu W, Huijun L, Zhe Z, Lixin J (2016). Outline of the report on cardiovascular diseases in China, 2014. Eur Heart J Suppl.

[2] Stevens W, Peneva D, Li JZ, Liu LZ, Liu G, Gao R, Lakdawalla DN (2016). Estimating the future burden of cardiovascular disease and the value of lipid and blood pressure control therapies in China. BMC Health Serv Res.

[3] GBD 2016 Causes of Death Collaborators (2017). Global, regional, and national age-sex specific mortality for 264 causes of death, 1980–2016: a systematic analysis for the Global Burden of Disease Study 2016. Lancet.

[4] Yang G, Wang Y, Zeng Y, Gao GF, Liang X, Zhou M, Wan X, Yu S, Jiang Y, Naghavi M (2013). Rapid health transition in China, 1990-2010: findings from the global burden of disease study 2010. Lancet..

[5] Liu Shiwei, Li Yichong, Zeng Xinying, Wang Haidong, Yin Peng, Wang Lijun, Liu Yunning, Liu Jiangmei, Qi Jinlei, Ran Sha, Yang Shiya, Zhou Maigeng (2019). Burden of Cardiovascular Diseases in China, 1990-2016. JAMA Cardiology.

[6] Li GX, Zhou B, Qi GX, Zhang B, Jiang DM, Wu GM, Ma B, Zhang P, Zhao QR, Li J (2017). Current trends for ST-segment elevation myocardial infarction during the past 5 years in rural areas of China's Liaoning Province: a multicenter study. Chin Med J.

[7] Li J, Li X, Wang Q, Hu S, Wang Y, Masoudi FA, Spertus JA, Krumholz HM, Jiang L (2015). ST-segment elevation myocardial infarction in China from 2001 to 2011 (the China PEACE-retrospective acute myocardial infarction study): a retrospective analysis of hospital data. Lancet..

[8] Zheng X, Curtis JP, Hu S, Wang Y, Yang Y, Masoudi FA, Spertus JA, Li X, Li J, Dharmarajan K (2016). Coronary catheterization and percutaneous coronary intervention in China: 10-year results from the China PEACE-retrospective CathPCI study. JAMA Intern Med.

[9] Song F, Yu M, Yang J, Xu H, Zhao Y, Li W, Wu D, Wang Z, Wang Q, Gao X (2016). Symptom-onset-to-balloon time, ST-segment resolution and in-hospital mortality in patients with ST-segment elevation myocardial infarction undergoing primary percutaneous coronary intervention in China: from China acute myocardial infarction registry. Am J Cardiol.

[10] Henan Municipal Bureau Statistics, NBS Survey Office in Henan (2015). Henan statistical yearbook 2015.

[11] Zhang Y, Gao C, Duan G, Liu X, Zhang H, Zhang C, Hu D (2015). Survey on the early reperfusion therapy status in patients with ST-segment elevation myocardial infarction hospitalized in tertiary and secondary hospitals in Henan province. Zhonghua Xin Xue Guan Bing Za Zhi.

[12] Zhang Y, Yang S, Liu X, Li M, Zhang W, Yang H, Hu D, Gao C, Duan G (2016). Management of ST-segment elevation myocardial infarction in predominantly rural Central China: a retrospective observational study. Medicine (Baltimore).

[13] Zhang Y, Huo Y (2011). Early reperfusion strategy for acute myocardial infarction: a need for clinical implementation. J Zhejiang Univ Sci B.

[14] Hao Y, Liu J, Liu J, Smith SJ, Huo Y, Fonarow GC, Ma C, Ge J, Taubert KA, Morgan L (2016). Rationale and design of the improving Care for Cardiovascular Disease in China (CCC) project: a national effort to prompt quality enhancement for acute coronary syndrome. Am Heart J.

[15] Xu H, Li W, Yang J, Wiviott SD, Sabatine MS, Peterson ED, Xian Y, Roe MT, Zhao W, Wang Y (2016). The China acute myocardial infarction (CAMI) registry: a national long-term registry-research-education integrated platform for exploring acute myocardial infarction in China. Am Heart J.

[16] Thygesen K, Alpert JS, Jaffe AS, Simoons ML, Chaitman BR, White HD, Katus HA, Lindahl B, Morrow DA, Clemmensen PM (2012). Third universal definition of myocardial infarction. Circulation..

[17] O'Gara PT, Kushner FG, Ascheim DD, Casey DJ, Chung MK, de Lemos JA, Ettinger SM, Fang JC, Fesmire FM, Franklin BA (2013). 2013 ACCF/AHA guideline for the management of ST-elevation myocardial infarction: executive summary: a report of the American College of Cardiology Foundation/American Heart Association Task Force on Practice Guidelines: developed in collaboration with the American College of Emergency Physicians and Society for Cardiovascular Angiography and Interventions. Catheter Cardiovasc Interv.

[18] Li X, Murugiah K, Li J, Masoudi FA, Chan PS, Hu S, Spertus JA, Wang Y, Downing NS, Krumholz HM, et al. Urban-rural comparisons in hospital admission, treatments, and outcomes for ST-segment-elevation myocardial infarction in China from 2001 to 2011: a retrospective analysis from the China PEACE study (patient-centered evaluative assessment of cardiac events). Circ Cardiovasc Qual Outcomes. 2017. 10.1161/CIRCOUTCOMES.117.003905. 10.1161/CIRCOUTCOMES.117.003905 PMC6312853 29158421

[19] Sun H, Yang YJ, Xu HY, Yang JG, Gao XJ, Wu Y, Li W, Wang Y, Liu J, Jin C (2016). Survey of medical care resources of acute myocardial infarction in different regions and levels of hospitals in China. Zhonghua Xin Xue Guan Bing Za Zhi..

[20] Medina HM, Cannon CP, Zhao X, Hernandez AF, Bhatt DL, Peterson ED, Liang L, Fonarow GC (2011). Quality of acute myocardial infarction care and outcomes in 33,997 patients aged 80 years or older: findings from get with the guidelines-coronary artery disease (GWTG-CAD). Am Heart J.

[21] Peiyuan H, Jingang Y, Haiyan X, Xiaojin G, Ying X, Yuan W, Wei L, Yang W, Xinran T, Ruohua Y (2016). The comparison of the outcomes between primary PCI, fibrinolysis, and no reperfusion in patients ≥ 75 years old with ST-segment elevation myocardial infarction: results from the Chinese acute myocardial infarction (CAMI) registry. PLoS One.

[22] Hao Y, Liu J, Liu J, Yang N, Smith SJ, Huo Y, Fonarow GC, Ge J, Taubert KA, Morgan L (2019). Sex differences in in-hospital management and outcomes of patients with acute coronary syndrome: findings from the improving Care for Cardiovascular Disease in China (CCC) project. Circulation..

[23] Du X, Spatz ES, Dreyer RP, Hu S, Wu C, Li X, Li J, Wang S, Masoudi FA, Spertus JA, et al. Sex Differences in Clinical Profiles and Quality of Care Among Patients With ST-Segment Elevation Myocardial Infarction From 2001 to 2011: Insights From the China Patient-Centered Evaluative Assessment of Cardiac Events (PEACE)-Retrospective Study. J Am Heart Assoc. 2016. 10.1161/JAHA.115.002157. 10.1161/JAHA.115.002157 PMC4802449 26903002

[24] Sutton NR, Li S, Thomas L, Wang TY, de Lemos JA, Enriquez JR, Shah RU, Fonarow GC (2016). The association of left ventricular ejection fraction with clinical outcomes after myocardial infarction: findings from the acute coronary treatment and intervention outcomes network (ACTION) registry-get with the guidelines (GWTG) Medicare-linked database. Am Heart J.

[25] Guan W, Venkatesh AK, Bai X, Xuan S, Li J, Li X, Zhang H, Zheng X, Masoudi FA, Spertus JA (2019). Time to hospital arrival among patients with acute myocardial infarction in China: a report from China PEACE prospective study. Eur Heart J Qual Care Clin Outcomes.

[26] Fu R, Song CX, Dou KF, Yang JG, Xu HY, Gao XJ, Liu QQ, Xu H, Yang YJ (2019). Differences in symptoms and pre-hospital delay among acute myocardial infarction patients according to ST-segment elevation on electrocardiogram: an analysis of China acute myocardial infarction (CAMI) registry. Chin Med J.

[27] Yang X, Zhao Y, Wu H, Yan M, Wang Y, Li Y, Guo X (2012). The coexistence of comorbidities at admission is an independent predictor of 30-day mortality of patients hospitalized with acute myocardial infarction: analysis of 5523 cases in China. Int J Cardiol.

[28] Fu R, Song C, Yang J, Wang Y, Li B, Xu H, Gao X, Li W, Liu J, Dou K (2018). CAMI-NSTEMI score- China acute myocardial infarction registry-derived novel tool to predict in-hospital death in non-ST segment elevation myocardial infarction patients. Circ J.

[29] Li X, Li J, Masoudi FA, Spertus JA, Lin Z, Krumholz HM, Jiang L (2016). China PEACE risk estimation tool for in-hospital death from acute myocardial infarction: an early risk classification tree for decisions about fibrinolytic therapy. BMJ Open.

