# Supplementary material for: Rationale and design of the Henan ST elevation myocardial infarction (STEMI) registry: a regional STEMI project in predominantly rural central China
Source: BMC Cardiovasc Disord. 2019 Nov 28;19:271. doi: 10.1186/s12872-019-1250-9 (PMC6883687; doi:10.1186/s12872-019-1250-9)
Supplement: Supplementary file 1 — Additional file 1. Full list of hospitals in the Henan STEMI registry [file 12872_2019_1250_MOESM1_ESM.docx]

**Appendix 1.** Full list of hospitals in the Henan STEMI registry

| **Hospital** | **Prefecture/City** | **PI** |
| --- | --- | --- |
| Zhengzhou University People's Hospital | Zhengzhou | Chuanyu Gao |
| Xinxiang Central Hospital | Xinxiang | Zhifang Wang |
| The First Affiliated Hospital of Henan Science and Technology University | Luoyang | Xuming Yang |
| The First People's Hospital of Shangqiu | Shangqiu | Shengli Li |
| Xiping County People's Hospital | Zhumadian | Hualing Xia |
| Zhumadian central hospital | Zhumadian | Baoqiang Bai |
| The People's Hospital of Yongcheng | Shangqiu | Changming Tian |
| Xihua County People's Hospital | Zhoukou | Jinbo Li and Chuntong Wang |
| Shangcai County People's Hospital | Zhumadian | Yi Yang |
| The People's Hospital of Gongyi | Zhengzhou | Yanhui Gao |
| The Third Affiliated Hospital of Xinxiang medical college | Xinxiang | Haiyan Sun |
| The Second People's Hospital of Xinxiang | Xinxiang | Jixia Yan |
| The First People's Hospital of Lingbao | Sanmenxia | Wanke Li |
| The People's Hospital of Xingyang | Zhengzhou | Hepng Li |
| The Second People's Hospital of Nanyang | Nanyang | Huading Zhou |
| The People's Hospital of Jiaozuo | Jiaozuo | Haijun Zheng |
| Ningling County People's Hospital | Shangqiu | Chuanqian Zhang |
| Puyang Oil Field General Hospital | Puyang | Hengliang Wang |
| Fengqiu County People's Hospital | Xinxiang | Dequan Jing |
| New area People's Hospital of Luoyang | Luoyang | Fengxian Lin |
| Mianchi County People's Hospital | Sanmenxia | Suxia Chen |
| Wen County People's Hospital | Jiaozuo | Xiaoli Ji |
| The First People's Hospital of Xinxiang | Xinxiang | Guiye Zhao |
| Pingyu County People's Hospital | Zhumadian | Wei Wei |
| The People's Hospital of Hebi | Hebi | Peng Liu and Yanggui Liu |
| Xiayi County People's Hospital | Shangqiu | Xiangyang Cheng |
| The People's Hospital of Qinyang | Jiaozuo | Xiaowen Ma |
| Tongxu County hospital of Chinese medicine | Kaifeng | Chengwen Zhang |
| Song County People's Hospital | Luoyang | Liangping Wang |
| The Third People's Hospital of Shangqiu | Shangqiu | Changgang Tong |
| The Second People's Hospital of Pingdingshan | Pingdingshan | Ling Zhang |
| Zhecheng County People's Hospital | Shangqiu | Zhenfu Zhao |
| The Second People's Hospital of Jiyuan | Jiyuan | Ruilu Xue |
| The Second People's Hospital of Mengjin County | Luoyang | Hengshan Wei and Haitao Wang |
| Yudong Hospital of the First Affiliated Hospital of Henan University of Chinese Medicine | Shangqiu | Haojie Xu |
| The People's Hospital of Yanshi | Luoyang | Huihui Lang |
| Xuchang County People's Hospital | Xuchang | Xianzhang Li |
| The First Affiliated Hospital of Henan University | Kaifeng | Qiwei Tang |
| Neixiang County People's Hospital | Nanyang | Jianbo Jia |
| The People's Hospital of Dengfeng | Zhengzhou | Hongxu Geng |
| Nanzhao County People's Hospital | Nanyang | Yuchun Li |
| Minquan County People's Hospital | Shangqiu | Yonghong Shi |
| Yuhzou City hospital of Chinese Medicine | Xuchang | Hongxia Zhang |
| Tongxu County People's Hospital | Kaifeng | Dongsheng Wang |
| The Second People's Hospital of Xiayi County | Shangqiu | Yonggang Liu |
| Nanshi Hospital of Nanyang | Nanyang | Linming Zhao |
| The Sixteenth People's Hospital of Zhengzhou | Zhengzhou | Qinghua Xu |
| Zhecheng County hospital of Chinese Medicine | Shangqiu | Xinling Deng |
| The Third People's Hospital of Luoyang | Luoyang | Lipeng Li and Suqin Chen |
| Sheqi County People's Hospital | Nanyang | Gang Liu |
| The People's Hospital of Wugang | Pingdingshan | Keqi Liang |
| Xinye County People's Hospital | Nanyang | Linwu Zhang |
| Weishi County People's Hospital | Kaifeng | Jiandang Jiang |
| Runan County People's Hospital | Zhumadian | Weitian Xiao |
| Yichuan County hospital of Chinese Medicine | Luoyang | Hongwen Zhang |
| Yucheng County People's Hospital | Shangqiu | Baofu Liu |
| Minquan County hospital of Chinese Medicine | Shangqiu | Chengxuan Pan |
| The People's Hospital of Jiaozuo Macun District | Jiaozuo | Na Sun |
| Qi County People's Hospital | Kaifeng | Shengke Zhu |
| Ye County People's Hospital | Pingdingshan | Jie Yang |
| Huangchuan County People's Hospital | Xinyang | Zheng Liu |
| Queshan County People's Hospital | Zhumadian | Yaoze Li |
| The Central Hospital of Yima Coal Industry Group CO. LTD | Sanmenxia | Fengyun Lu |
| Suiping County People's Hospital | Zhumadian | Qingchun Zhang |
| Fangcheng County People's Hospital | Nanyang | Yintao Qiao |
| The Second People's Hospital of Xichuan County | Nanyang | Lianjie Li |
